# Perspectives of La_0.9_Sr_0.1_Fe_0.9_Co_0.1_O_3±*δ*_ perovskite obtained by Pechini and sonochemical methods: a case study

**DOI:** 10.1098/rsos.240627

**Published:** 2024-12-11

**Authors:** Gloria E. de la Huerta-Hernández, Tatiana Rodríguez-Flores, Armando Reyes-Montero, Iván Castro-Cisneros, Isaías Hernández-Pérez, José A. Chávez-Carvayar

**Affiliations:** ^1^ Departamento de Ciencias Básicas, Universidad Autónoma Metropolitana-Azcapotzalco, Av. San Pablo 180, Col. Reynosa Tamaulipas, Ciudad de México 02200, Mexico; ^2^ Instituto de Investigaciones en Materiales, Universidad Nacional Autónoma de México, Ciudad Universitaria, Ciudad de México 04510, Mexico; ^3^ Facultad de Ingeniería, Universidad Autónoma del Carmen, Av. Central s/n esq. Fracc. Mundo Maya, Ciudad del Carmen 24115, Mexico

**Keywords:** ultrasonic synthesis, LSFC perovskite, SOFC

## Abstract

The sonochemical method is a novel synthesis route that takes advantage of the use of ultrasonic radiation to obtain different nanomaterials with an improvement in the process variables and material characteristics. In this work, two different synthesis routes to obtain a double perovskite structure were compared in detail. The Pechini synthesis method is a widely used and effective way to obtain this kind of structure by the formation of a cross-linked network of metal cations. Alternatively, in sonochemistry, chemical reactions occur with the application of powerful ultrasound radiation. The final characteristics of synthesized powders of La_0.9_Sr_0.1_Fe_0.9_Co_0.1_O_3±_*_δ_* (LSFC) were analysed thermally, structurally, morphologically and optically, and their transport properties were evaluated to determine their semiconductor character in a solid oxide fuel cell device and in photocatalytic processes. Structural results indicated a well crystallized perovskite structure with a single cubic phase. The cell parameter *a* (approx. 3.89 Å) and crystal size measurements (11–20 nm) were determined by Rietveld refinement for samples obtained by both synthesis methods. Thescanning electron microscope micrographs showed aggregates with homogeneous morphology and a uniform particle size distribution, with a rough and porous surface. Optical properties were determined by ultraviolet–visible spectrophotometry and photoluminescence, resulting in an *E*_g_ of 1.2 and 0.8 eV in samples from Pechini and sonochemistry, respectively.

## 1. Introduction

The adverse effects of climate change because of greenhouse gas emissions and global energy needs are becoming increasingly worrying. Therefore, some efforts to solve these problems are focusing on the creation of environmentally friendly energy sources. In this context, fuel cells are an attractive energy conversion technology that has gained much attention owing to growing global energy requirements.

Perovskite oxides, with a general formula ABO_3_, are considered among all promising materials owing to the flexibility and adaptability of their physical, chemical and catalytic characteristics. Basically, their structural properties are modified by the substitution of the A and/or B sites of the perovskite [1]. Moreover, any change in the occupancy equivalence at the A and/or B sites will result in the stabilization of the unstable oxidation state of the transition cations at the B site, causing a change in the electronic structure and catalytic performance [2]. This property makes oxide perovskites an optimal choice for oxygen reduction reactions and oxygen evolution reactions in an alkaline medium [3].

Ternary perovskite compounds have been produced by a wide collection of syntheses for different electroceramic equipment purposes, including electromechanical, electro-optical and electronic tools [4]. Lanthanum-based perovskites, for example, have been proven in different applications for catalytic, electronic, optical and luminescence devices [5]. The incorporation of La improves the conductivity in the ceramic material by increasing the reactive sites and generating vacancies. This characteristic promotes the ionic flow in the crystal lattice [6]. The most studied single phases are LaMnO_3_ [7], LaFeO_3_ [8] and LaCoO_3_ [9]; solid solutions include La*_x_*Sr_1-*x*_MnO_3±*δ*_ (LSM) [10], La*_x_*Sr_1-*x*_FeO_3±*δ*_ (LSF) and La*_x_*Sr_1-*x*_CoO_3±*δ*_ (LSC) [11]. Currently, other combinations, such as La*_x_*Sr_1-*x*_Mn*_y_*Cr_1-*y*_O_3±*δ*_ (LSMCr), La*_x_*Sr_1-*x*_Fe*_y_*Co_1-*y*_O_3±*δ*_ (LSFC) and La*_x_*Sr_1-*x*_Mn*_y_*Co_1-*y*_O_3±*δ*_ (LSMC), show a suitable mix of ionic/electronic properties and interesting catalytic activity towards hydrogen peroxide [12] and oxygen reduction.

In particular, LaFeO_3_-based perovskites have high structural stability, which allows their use in fuel cells [13,14], as gas sensors [15] and for photocatalytic applications [16,17], owing to their structural, morphological and optical properties. However, as the A and/or B sites are substituted with low concentrations of Sr and Co cations, respectively, the crystal structure preserves symmetry, the band gap value decreases, and oxygen vacancies increase. Owing to the nature of perovskite-type materials, several synthesis routes have been studied for their preparation (e.g. solid-state synthesis, sol–gel, combustion, coprecipitation) [18]. Among these synthesis methods, the Pechini method is an integrated process suitable for obtaining single-phase nanometric products with good control of structure, crystal size, stoichiometry, morphology and porosity. Furthermore, this technique operates under environmental pressure, making it one of the main routes for the synthesis of perovskite-based ceramics.

In double-perovskite-type oxides, with chemical formula A’A’’B’B’’O_6_, B’-O_6_ and B’’-O_6_ octahedra are arranged alternately by means of corner-sharing, with A cations occupying the voids between the octahedra [19]. Unlike a single-perovskite-type oxide, the catalytic performance of double-perovskite-type oxides can be effectively improved by the synergy and coordination of the metals in the special structure of B’-O-B’’ [20,21]. In addition, the A-site cations have an important role in controlling the valence state of the B-site metals and stabilizing the structure, simultaneously causing lattice defects [22]. As a result, a different configurations and exchange interactions between A’/A’’ and B’/B’’ ions can promote extensive use of these materials: double-perovskite (La,Sr)(Co,Fe)O_3-δ_ ceramics, owing to their important ionic and electronic conductivity, as well as their high oxygen catalytic activity, are optimum candidates for solid oxide fuel cell (SOFC) technology [23,24].

**Figure 1.**
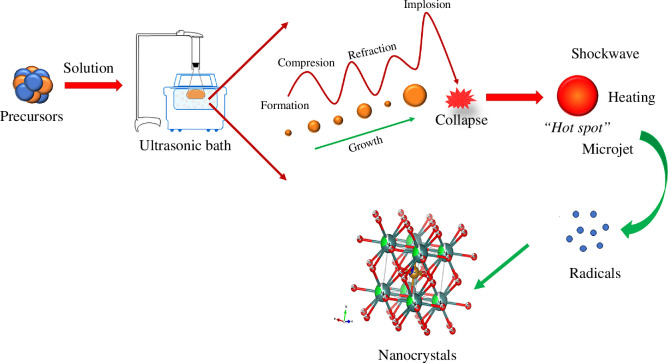
Schematic representation of the sonochemistry process for the synthesis of perovskite materials.

In recent decades, the use of ultrasonic waves to obtain nanomaterials has opened a novel chemical route for the synthesis of perovskites; the use of ultrasound waves to fabricate a variety of nanostructures is an area of interest to many scientists because it has significant benefits [25–27]. Currently, the sonochemical method has been employed for the preparation of metal carbonyl compounds, metal colloids, polyheteronuclear group complexes, metal oxides (including TiO_2_, Fe_3_O_4_, ZnO) and other complex compounds [28], where ultrasonic radiation accelerates reactions because of a high temperature/pressure medium, causing reactions to occur in a short time [29,30]. This synthesis method is based on the principle of acoustic cavitation, where the formation, growth, and implosive collapse of nanobubbles in a liquid create extreme conditions within the collapsing nanobubble and serve as the origin of most sonochemical phenomena (see figure 1). Furthermore, low energy consumption, good ability to tune dimensions, architecture and morphology of different compounds, and simplicity are other unique features of using sonochemistry [31–33].

In particular, sonochemistry has been used to determine whether nanostructures possess new size-dependent properties, such as electrical, magnetic, mechanical, optical and chemical properties, that differ greatly from those of bulk materials. Considering that the reaction times required in conventional methods are usually several days, the drastic reduction produced by sonochemistry synthesis will result in fast production of ceramic material [32,34].

In this work, a single-phase nanocrystalline porous compound La_0.9_Sr_0.1_Fe_0.9_Co_0.1_O_3±*δ*_ (LSFC) was successfully obtained using Pechini and sonochemical synthesis methods. Thermal, structural, morphological, and optical characterization were performed by X-ray diffraction (XRD), scanning electron microscopy (SEM), atomic force microscopy (AFM) and high-resolution transmission electron microscopy (HRTEM). Ultraviolet–visible (UV–Vis) spectrophotometry and photoluminescence (PL) spectroscopy were used to evaluate and correlate transport properties obtained by impedance spectroscopy (IS). This work shows the advantages of using the sonochemistry process over a traditional synthesis method to produce La-based perovskite materials.

## 2. Material and methods

The starting materials were La(NO_3_)_3_·6H_2_O (Sigma-Aldrich, 99.99%), Sr(NO_3_)_2_ (Sigma-Aldrich, ≥99%), Fe(NO_3_)_2_·9H_2_O (Sigma-Aldrich, ≥98%) and Co(NO_3_)_3_·6H_2_O, (Sigma-Aldrich, ≥98%). In addition, citric acid (Sigma-Aldrich, ≥99.5%), ethylene glycol (J. T. Baker, ≥99%) and NH_4_OH (J. T. Baker, 28–30%) were used for the Pechini method. For the sonochemistry method, methanol (J. T Baker, 99.9%) and acetone (J. T. Baker, 99.7%) were used as solvents.

### 2.1. Pechini synthesis

**Figure 2.**
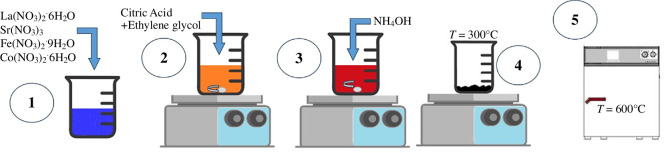
Schematic representation of the Pechini synthesis.

Figure 2 displays the following Pechini synthesis method to produce LSFC ceramic powders. First, all precursors were dissolved in deionized water (step 1); see electronic supplementary material, table S1 for the quantities of precursors used. After complete dissolution, citric acid (0.0378 mol) was added as a chelating agent to initiate sol formation, followed by the addition of ethylene glycol (0.0567 mol) employed for the separation of the nucleation centre of the ceramic powders (step 2). Then, NH_4_OH (12 ml) was added dropwise to the mixture. The solution had an initial pH of 1 and presented four colour changes with different pH values as NH_4_OH volume increased. This was an adequate guide to reach pH 7 (step 3) [35]. Temperature was increased to 300°C for 2 h to obtain the amorphous oxide (polymeric resin) and surface water began to evaporate (step 4). Finally, the sample was calcined at 600°C for 2 h (step 5).

### 2.2. Sonochemistry method

**Figure 3.**
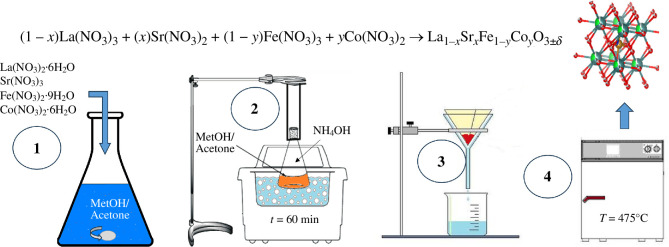
Schematic representation of the sonochemistry process.

Figure 3 presents the sonochemistry synthesis process used to obtain LSFC ceramic powders. First, nitrates were dissolved in a methanol/acetone solution (25 ml of each solvent) under continuous stirring (step 1). The obtained solution was immersed in an ultrasonic bath (Fischer Scientific, FS20 with an operating frequency of 40 kHz and 80 W) and sonicated for 50 min (step 2). Then, after 30 min, ethylene glycol (0.0126 mol) was added to disperse the ceramic powders obtained. After sonication, precipitation was initiated using NH_4_OH diluted 1 : 1 with ionized water (5 ml of each) until pH 7 was reached. The precipitate was filtered and dried at 25°C for 24 h (step 3). Finally, the powders were heated at 475°C for 2 h in a high-temperature furnace (step 4).

Thermogravimetric (TG) measurements were performed on prepared samples in an SDT Q600 SDG TG analyser with a heating ramp of 10°C min^−1^, from 25 to 800°C (under air atmosphere). Weight loss (%) and thermal stability of the samples were calculated using Instrumental Universal Analysis 2000 software, v. 4.5 (TA Instruments). X-ray powder diffraction (XRD) measurements were performed on calcined ceramic powders on a Bruker D8 Advance diffractometer (CuKα radiation *λ* = 1.5406 Å) in the range 20–120° (2*θ* scale) with a step size of 0.02° and a collection time of 5 s per step. The results were analysed using Diffract Suite software and compared with standards from the ICDD database (PDF 01−075−0439). The FullProf Suite program was used to perform Rietveld refinements [36].

Two methods were used to determine the size of the crystals; the first was the Scherrer method [37]:


(2.1) $$D=\frac{k\lambda }{\beta_{hkl}\;cos\theta }.$$

The second was a graphical method, using the Williamson–Hall plot, where the full-width half maximum (FWHM) and Rietveld refinement values were used to calculate *D*, in equation (2.2). One of the main advantages of this method is that it considers that the peak broadening is given by the crystal and the deformation effects within the crystals:

(2.2) $$\beta_{hkl}cos\theta =\left(\frac{k\lambda }{Dcos\theta }\right)+\left(4\varepsilon sin\theta \right),$$

where, for both equations, *β_hkl_* is the width of the peak at half-maximum intensity, *θ* is the position of the peak, *D* is the average crystal size, *λ* is the wavelength of the X-rays, *k* is a constant equal to 0.9 and *ε* ≈ *β*/tan*θ*. The term *β_hkl_* cos*θ* was plotted against 4ε sin*θ*, and the slope and *y*-intercept of the fitted line represent the strain and particle size, respectively.

High-resolution transmission electron microscopy (HRTEM) micrographs of synthesized ceramic powders were obtained on a Titan FEI microscope with a resolution of 2.0 nm and a voltage of 300 keV. For the analysis of the micrographs, Digital Micrograph (MT) software, v. 3.7.0, was used. The Raman spectra were measured in a Raman Renishaw IN Via with laser wavelength of 532 nm (green laser), the spectra measurements were carried out with five accumulations for 5 s for each one and 50% power. The size and morphology of the obtained powders were analysed using a JEOL 7600F field emission scanning electron microscope with 2.3 Å resolution, 50 000× amplification, 10 kV acceleration voltage and secondary electron detector. Atomic force microscopy (AFM) was performed on a JEOL JSPM-421 microscope; samples were prepared in thin slices and micrographs were obtained in tapping mode. N_2_ adsorption isotherms were carried out on a Micromeritics ASAP 2020. Experiments were performed in a liquid nitrogen bath at local atmospheric pressure (*ca* 77.981 kPa) for N_2_. A Micromeritics Dewar chiller and a Neslab RTE7 refrigerated bath were combined to ensure cryogenic conditions. Samples were degassed for 1 h at a pressure below 1 × 10^−5^ bar. After degassing, the sample tube was refilled with dry nitrogen and transferred to the analysis port, and evacuation continued for another 12 h at room temperature. The free volume was then measured with helium, and samples were degassed again for 12 more hours at room temperature, before recording the N_2_ adsorption isotherms. The optical band gap (*E*_g_) and Urbach energies (*E*_U_) were calculated from UV–Vis spectroscopy, performed on a Varian Cary I spectrophotometer, equipped with a DRA-CA-30I integration sphere, from 900 to 190 nm. To calculate *E*_g_ and *E*_U_, the Tauc equation and the Urbach diagram were applied. The optical band gap can be described for the Tauc model:


(2.3) $$\alpha h\nu =A{\left(h\nu -E_{\text{g}}\right)}^{m}$$

where *α* is the absorption coefficient*, hν* is the energy of the photon, *A* is a proportionality constant, *E*_g_ is the optical band gap, and *m* is related to sample electronic transitions and can take the value 1/2 or 2, for the direct and indirect transition, respectively.

The photoluminescence spectrum was captured on a Varian Cary Eclipse equipped with a xenon lamp, using an excitation wavelength of 330 nm and an emission range of 340 to 600 nm. For impedance spectroscopy (IS) characterization of LSFC materials, ceramic samples were ground for 30 min, pressed into pellets and sintered at 1400°C for 2 h. The sintered pellets were polished to obtain smooth faces. IS measurements were carried out on a Solartron SI-1260 connected to a 1296 dielectric interface (frequency range from 1Hz to 10 MHz; 10 points per decade and 100 mV) over a temperature range of 25 to 300°C. The acquired impedance results were analysed using ZView software.

## 3. Results and discussion

### 3.1. Thermal properties

**Figure 4.**
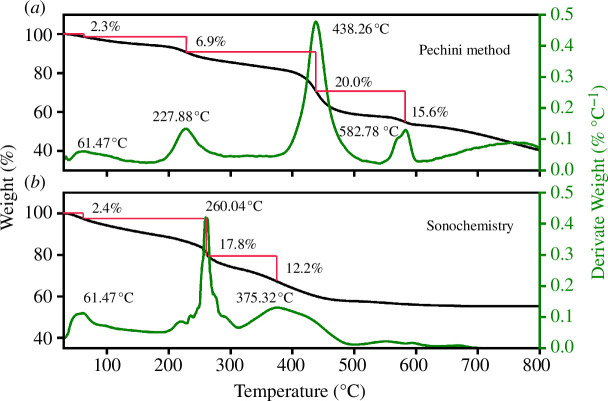
Thermogravimetric analysis (TGA) and first derivative of TGA curves as a function of temperature, for samples synthesized by (*a*) Pechini method and (*b*) sonochemistry.

Figure 4 shows the TGA and first derivative TGA for La_0.9_Sr_0.1_Fe_0.9_Co_0.1_O_3±*δ*_ samples obtained by the Pechini method and sonochemistry. It is observed that in both synthesis methods, the first process (approx. 61°C) is associated with the loss of surface molecular water. The second process in figure 4*a*, at about 228°C, is attributed to the decomposition of the remaining ethylene glycol and citric acid. A third mass loss (approx. 20%), located near 438°C, is related to the calcination of nitrates from the precursors. Finally, the last process that occurs, at approximately 583°C, is where the formation of the crystalline phase begins. It has been reported that the optimum temperature range where nano LaFeO_3_ ceramics are obtained lies between 400 and 500°C [38]. Therefore, at 583°C it is possible to obtain a lower grain size of the LSFC, with no secondary phases.

For the samples obtained by sonochemistry (figure 4*b*), a narrowed exothermic peak, observed at approximately 260°C, is associated with the decomposition of the precursor nitrates. Unlike the Pechini method, this process occurs with a temperature difference of 178°C. The last mass loss detected, at approximately 375°C, is related to the initial stage of the crystalline phase formation. Above this temperature, no mass changes were observed. The conditions produced by the sonochemistry method favour the synthesis of LSFC samples with a low thermal treatment. The calcination temperature was set considering not only temperature, but also the time spend in the furnace, which allowed the formation of the crystalline phase [39]. In sonochemistry, the calcination temperature took into account the same parameters: TGA temperature, time in the furnace and synthesis route to obtain the crystalline phase. Moreover, when the calcination temperature decreases, the crystal size is smaller, increasing the surface area.

### 3.2. Structural analysis

#### 3.2.1. X-ray diffraction

**Figure 5.**
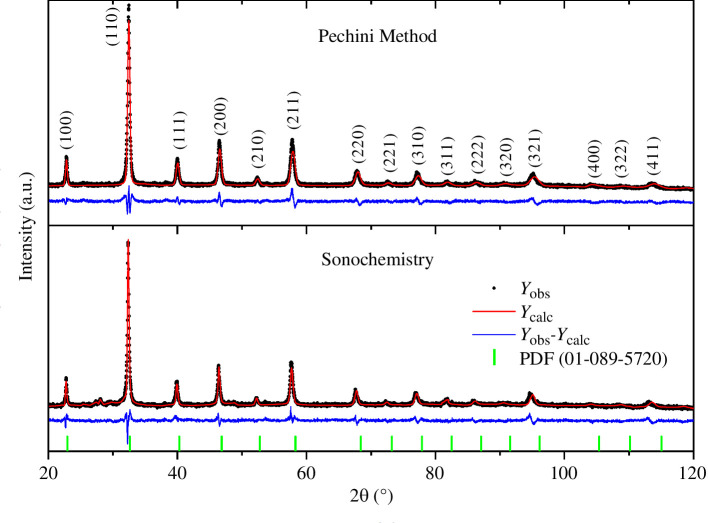
Rietveld refinement of X-ray diffraction patterns of calcined La_0.9_Sr_0.1_Fe_0.9_Co_0.1_O_3±*δ*_ ceramic powders obtained by the Pechini method and sonochemistry.

The X-ray diffraction (XRD) Rietveld refinement of the calcined LSFC ceramic powders, obtained by Pechini and sonochemistry, is shown in figure 5. According to the XRD results, the LSFC phase was corroborated and indexed; the main peaks are located at 32.4° (1 1 0), 46.5° (2 0 0) and 57.6° (1,1,2) and correspond to a cubic symmetry. For both synthesis routes a single crystalline phase was obtained. The main structural refined parameters such as lattice parameters, cell volume and stoichiometry in the unit cell are shown along with the quality factor (*R*_wp_, *R*_exp_ and *χ*^2^) in table 1. Moreover, the small signal between 20–30° is related to a Kβ signal produced by the diffractometer monochromator.

**Table 1.** Refined structural parameters and quality factor for La_0.9_Sr_0.1_Fe_0.9_Co_0.1_O_3±*δ*_ (LSFC) ceramic powders using a $Pm\bar{3}m$ cubic phase model.

	Stoichiometry at the unit cell					Structural parameters					
	La	Sr	Fe	Co	O	*a* (Å)	*V* (Å^3^)	(g cm^−3^)	*R*_wp_	*R*_exp_	*χ* ^2^
Pechini method	0.85	0.16	0.84	0.15	2.7	3.897 [1]	59.20 [4]	6.42	6.26	3.32	3.57
sonochemistry	0.89	0.11	0.89	0.11	2.55	3.905 [5]	59.57 [9]	6.53	8.04	3.86	4.34

**Figure 6.**
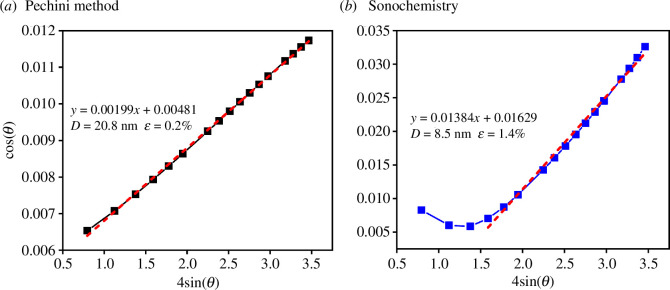
Williamson–Hall plot for La_0.9_Sr_0.1_Fe_0.9_Co_0.1_O_3±*δ*_ ceramic powder obtained by (*a*) Pechini and (*b*) sonochemistry methods, where D = crystal size, ε = microdeformation (%) and θ = the peak position.

From the FWHM results obtained by Rietveld refinement for both LSFC samples, the Williamson–Hall (W–H) plot showed a positive slope, indicating a slight deformation due to the small increase in the lattice size. The crystal size (D) calculated using the Scherrer equation and the W–H plot for the sample synthesized by the Pechini method was approximately 21 nm (figure 6*a*). A smaller crystal size (approx. 9 nm) was obtained for the sonochemistry samples (figure 6*b*). It seems that acoustic cavitation limits particle growth owing to the high pressure and temperature created around the nanobubbles [40], favouring crystallinity and a single-phase formation.

Furthermore, the deformation of the crystal lattice was determined using the W–H graphs, where the sample obtained by the sonochemical method showed a difference of 1.4% compared with the 0.2% of the Pechini sample. This result can be attributed to the higher level of energy generated by the ultrasonic waves used in the synthesis, resulting in a higher number of defects.

#### 3.2.2. High-resolution transmission electron microscopy

**Figure 7.**
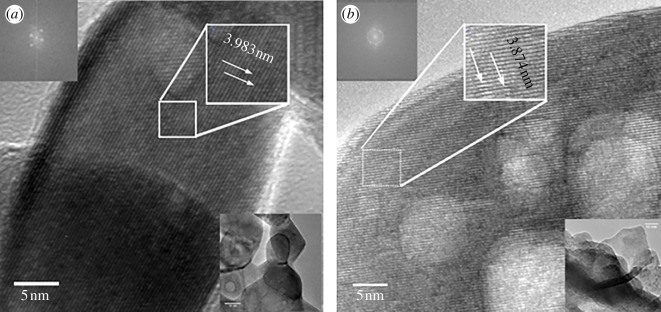
Cell parameters determined by high resolution transmission electron microscopy of La_0.9_Sr_0.1_Fe_0.9_Co_0.1_O_3±*δ*_ compounds synthesized by (*a*) the Pechini method and (*b*) sonochemistry.

HRTEM micrographs confirmed the crystallographic parameters of the LSFC samples. For the sample synthesized by the Pechini method, an average interplanar distance of 3.893 Å was determined (figure 7*a*), which is close to the value of 3.897 Å obtained by XRD for the (1 0 0) plane. The micrographs showed a crystal size of around 20 nm, a value comparable to those determined by the Scherrer equation and the W–H plot (20.8 nm).

The sonochemical ceramic powder showed an average interplanar distance of 3.874 nm (figure 7*b*), determined for the (1 0 0) plane. This value is comparable to that obtained by Rietveld refinement (3.905 nm). The average crystal size observed is 10 nm, which agrees with that obtained by the methods of Scherrer and W–H.

#### 3.2.3. Raman spectroscopy

**Figure 8.**
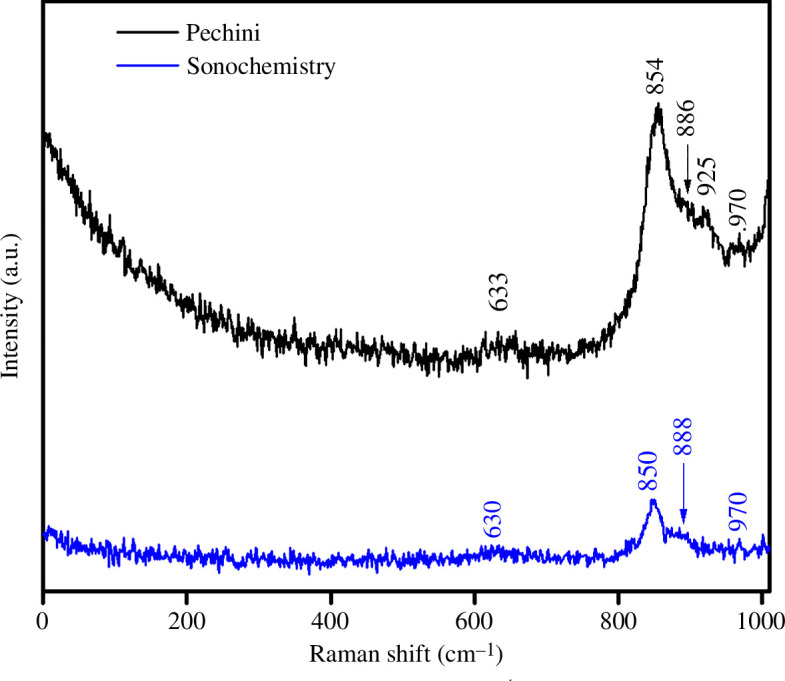
Raman spectra of the ceramic powders synthesized by the Pechini method (black line) and sonochemistry (blue line).

Raman spectra of the LSFC powders (figure 8) were collected at room temperature to corroborate the crystallinity and purity of the samples. The results show peaks below the values of 1000 cm^–1^, corresponding to double-perovskite materials [41]. In addition, no modal vibration can be observed in the region of 200–500 cm^–1^; these peaks were associated with single-perovskite structures. The peak located around 630 cm^−1^ is attributed to the *E*_g_ vibrational mode [42], while the peaks corresponding to stretching vibrations of the B‒O site bonds appear between 700 and 900 cm^–1^ [43]. It is worth noting that the vibration modes around 888 cm^–1^ in the sonochemical sample are split into two modes in the Pechini sample; this can be attributed to the degree of crystallization of LSFC because the Pechini sample shows higher particle sizes with respect to the sonochemical samples and better-ordered crystal structures [44], which agrees well with the XRD analyses.

### 3.3. Morphological analysis

#### 3.3.1. Scanning electron microscopy

**Figure 9.**
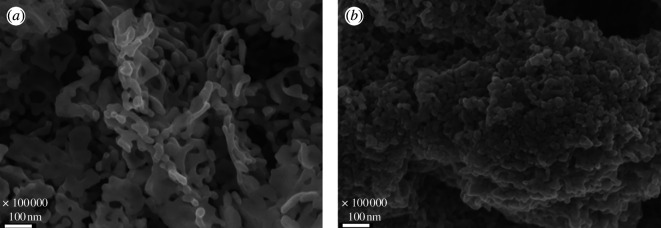
Scanning electron microscopy micrographs of the ceramic powders synthesized by (*a*) the Pechini method and (*b*) sonochemistry.

The influence of the synthesis method over the microstructure of the synthesized ceramic powder was observed by SEM. For the Pechini sample, a homogeneous distribution of a porous structure was identified, with a pore length of 16.34 nm and an area of 34.17 nm^2^ (figure 9*a*). The LSFC powder obtained by sonochemistry (figure 9*b*) shows a porous material that forms channels and large networks of agglomerated grains, resulting in an average value of 29.78 nm and 61.47 nm^2^ for pore length and area, respectively.

The morphological results for the La_0.9_Sr_0.1_Fe_0.9_Co_0.1_O_3±*δ*_ (LSFC) phase indicated that both methods are viable to obtain porous crystalline single-phase compounds. Although the sample synthesized by the Pechini method showed a greater number of pores, the sample obtained by sonochemistry presented a better definition of the channels; in particular, the stacking of the grains presented an interesting morphology. These channels could allow the flow of different gases within the solid, which is an important and desirable property for the different environmental applications of perovskites (e.g. power generationr and photocatalysis).

#### 3.3.2. Atomic force microscopy

**Figure 10.**
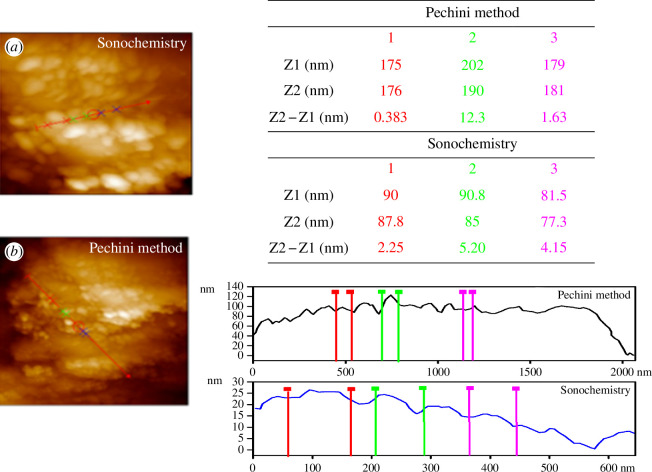
Atomic force microscopy micrographs and perfilometry of samples synthesized by (*a*) sonochemistry and (*b*) the Pechini method.

Additional morphological studies were carried out using AFM to determine the shape and size of the particles. The AFM micrographs showed spherical shapes with an average grain size of 86 nm for the homogeneously distributed Pechini sample (figure 10*b*). For the sample obtained by sonochemistry, grains with spherical shape and homogenous distribution were observed, with an average size of 49 nm (figure 10*a*).

The perfilometric results reveal that the powder obtained by sonochemistry showed smaller particles [45]. This effect is associated with the implosion of the nanobubbles; the ultrasonic waves prevent the growth of the grains by dispersion in the solvent and increase the amounts of the grain boundaries.

#### 3.3.3. Superficial area

**Figure 11.**
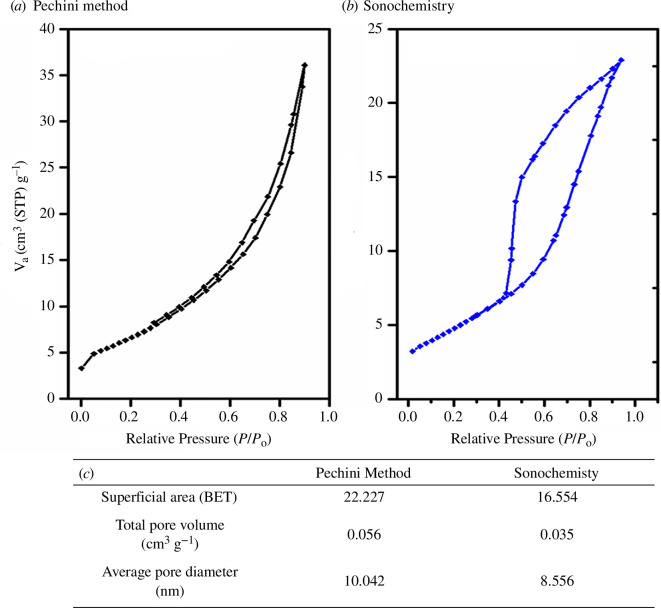
N_2_ desorption isotherm obtenied for samples synthesized by (*a*) Sonochemistry and (*b*) Pechini method. (*c*) Evaluated data calculated from the isotherms. V_A_= Volume adsorbed and *P*/*P*_0_ = relative pressure.

The N_2_ desorption isotherm (figure 11) was used to determine the pore size distribution by the Brunauer–Joyner–Halenda (BJH) method. For this purpose, spherical pores were considered, where both samples presented adsorption–desorption curves belonging to IVa. For the sample synthesized by the Pechini method, micropores are present in the relative pressure range 0.0–0.2, while for the sonochemical sample there is no evidence of micropores (figure 11c). The mesopore region can be observed in both samples, where the hysteresis loop is associated with capillary condensation in the desorption process in the range 0.3 < *P*/*P*_0_ < 0.9 (Pechini) and 0.4 < *P*/*P*_0_ < 0.9 (sonochemistry) and may be associated with pores formed between particles.

### 3.4. Optical analysis

#### 3.4.1. UV–Vis spectroscopy

**Figure 12.**
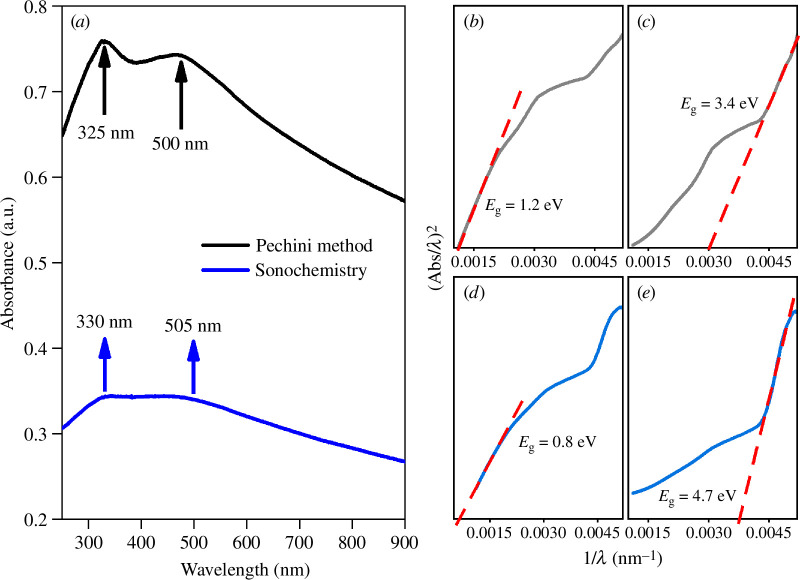
UV–visible spectra of La_0.9_Sr_0.1_Fe_0.9_Co_0.1_O_3±*δ*_: (*a*) optical absorbance, (*b*) band gap (*E*_g_) for the direct transition band of the Pechini sample, (*c*) *E*_g_ for the indirect transition band of the Pechini sample, (*d*) *E*_g_ for the direct transition band of the sonochemistry sample, and (*e*) *E*_g_ for its indirect transition band.

UV–Vis measurements showed that the samples obtained from both synthesis routes have an absorption edge around 325–330 nm; this type of perovskite absorbs in the UV region of the electromagnetic spectrum. A second absorption edge was also identified around 500 nm in the visible light region (figure 12*a*); however, the presence of a broad band located around 480 nm indicates that the samples synthesized by the Pechini method or sonochemistry absorb in the visible region, making them suitable for photocatalytic applications. The adsorption edge in the UV region is constant for both samples and the adsorption edge in the UV region was attributed to the electronic transition from the 2p orbitals forming the valence band to the 3d Fe orbitals in the conduction band [46].

The optical band gap (*E*_g_) was estimated from the intersections of the tangent to the graphs of the direct and indirect transition. These values were estimated by extrapolating the linear part of the curves obtained from the modified Kubelka–Munk function. The band gaps of the direct transitions showed a typical value for a semiconductor in LSFC, with 1.2 eV for the Pechini sample and 0.8 eV for the sonochemistry sample (figure 12*b*,*c*). For the indirect band gap transitions, the *E*_g_ value increases for both LSFC samples, 3.42 eV for the Pechini sample and 4.7 eV for the sonochemistry sample, corresponding to an insulating material (figure 12*d*,*e*). In the case of doubly substituted perovskites, the value of the band gap can be associated with a direct or indirect transition, depending on the path of the energy necessary to excite an electron from the valence band to the conduction band. Normally, in a semiconductor, in the band gap with direct transitions the position of the maximum of the valence band coincides with the minimum of the conduction band, but in indirect transitions the energy and momentum (wave vector) are displaced [47]. From the results in LFSC samples, the indirect transitions for the sample obtained by sonochemistry showed an increase in the *E*_g_ value wing to its smaller grain size. However, when *E*_g_ is calculated for direct transitions, the band gap value is higher for the sample synthesized by the Pechini method. To determine the value of band gap, the results suggest that LaFeO_3_ will behave as a direct band gap semiconductor because the electrons are more likely to have a direct transition than an indirect one; from these results, it is possible to consider LSFC as a ceramic semiconductor with direct transitions [48].

From UV–Vis measurements, the Urbach energy was calculated. In general, the Urbach energy and the band gap show an inverse relationship in their values, from which it can be observed that both samples show a very high disorder, where the sample synthesized by sonochemistry (4604 meV) has a higher disorder compared with the sample obtained from the Pechini method (4170 meV). This feature is attributed to the difference in the crystal size (9 nm for sonochemistry and 21 nm for Pechini), since the Urbach energy is inversely proportional to the crystallinity (see electronic supplementary material, figure S1). The values of the band gap and the Urbach energy are important because they allow establishment of the crystallinity and the nature of the defects of this perovskite structure, making it suitable in SOFC, catalysts and other applications [49,50].

#### 3.4.2. Photoluminescence spectroscopy

**Figure 13.**
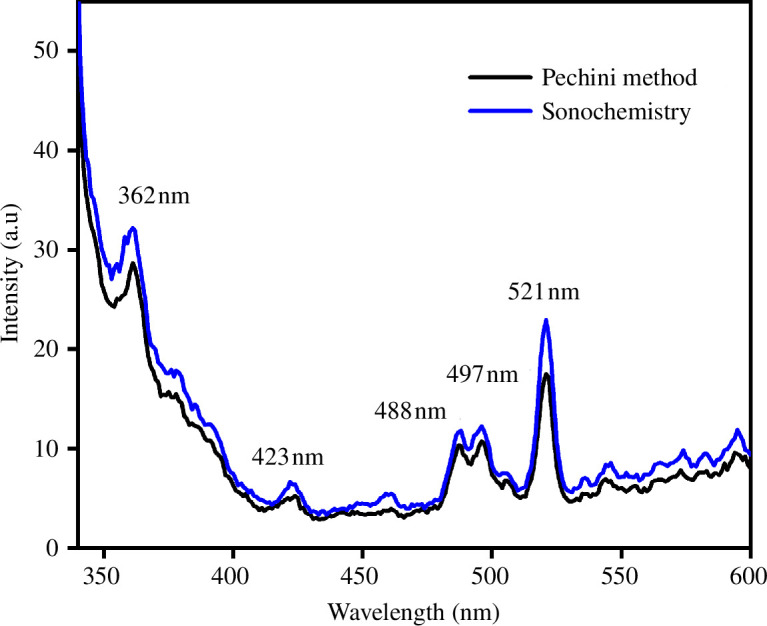
Photoluminescence spectrum of La_0.9_Sr_0.1_Fe_0.9_Co_0.1_O_3±*δ*_ for samples synthesized by the Pechini and sonochemistry methods.

The study of the emissions and defects in the perovskites synthesized by both methods is shown in figure 13. Both samples showed the same emission bands, but with different intensities, which reveals that the synthesis method does not generate different defects in the La_0.9_Sr_0.1_Fe_0.9_Co_0.1_O_3±*δ*_ perovskite. The band located at 362 nm is assigned to free-exciton emissions related to the recombination of exciton–exciton collisions. The photoluminescence (PL) signals at approximately 420 and 480 nm are mainly attributed to the electronic transitions from the conduction band to the valence band [16,51]. The bands between 488 and 497 nm are related to intrinsic defects such as the interstitial sites of the crystals [52,53]. Finally, the presence of oxygen vacancies is observed at 521 nm (green emission) [54,55]. The PL intensity specifies the content of surface oxygen vacancies and crystalline defects of the samples, with the sample with the highest PL intensity being the one with the most crystalline defects and oxygen vacancies, according to the Rietveld refinement stoichiometry results.

These results agree with those calculated from the optical properties and XRD results, where the sample with the smallest crystal size has a higher Urbach energy and therefore a higher PL intensity. According to this, as shown in figure 13, the perovskite synthesized by sonochemistry has more defects than that synthesized by the Pechini method. Lower luminescence emission intensity indicates a lower recombination rate caused by energy loss, resulting in better photocatalytic performance [56]. This characteristic was also observed using the W–H graph and the Urbach energy, where it was seen that the sample that presented more defects was that obtained by sonochemistry. This type of defect can be favourable for photocatalytic applications under visible and UV light.

### 3.5. Transport properties

**Figure 14.**
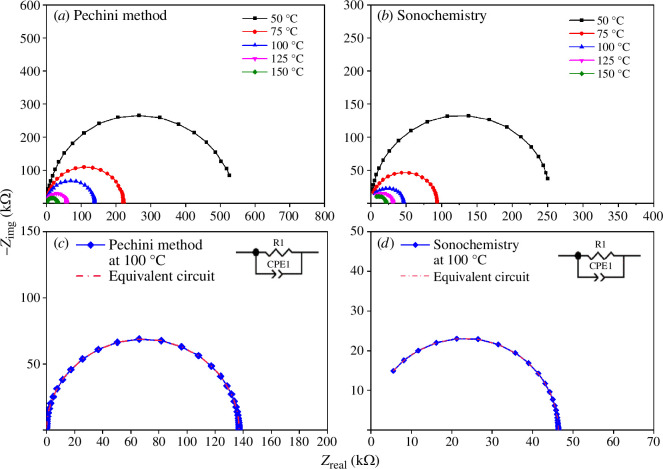
(*a*) Complex Impedance plots and (*b*) equivalent circuit of La_0.9_Sr_0.1_Fe_0.9_Co_0.1_O_3±*δ*_ obtained by the Pechini method. (*c*) Complex Impedance plots and (*d*) equivalent circuit for the sonochemistry sample. The Real (Zreal) and Imaginary (-Zimg) part of the impedance was evaluated in a frequency range from 1 Hz to 10 MHz. The obtained complex impedance plot at 100°C was simulated using a resistor (R) and a constat phase element (CPE).

The impedance spectroscopy technique was used to evaluate the transport properties of the samples and to determine the activation energy (*E*_a_). Complex impedance measurements as a function of temperature were performed on sintered samples, and the results are shown as Nyquist plots (figure 14). For the samples obtained by both synthesis methods, a single incomplete semicircle was observed. For the LSFC Pechini sample, the response is lost above 300°C (see electronic supplementary material, figure S5). However, the signal for the sample synthesized by sonochemistry disappears around 150°C (figure 14*c*). This effect is promoted by inductance as it begins to dominate over the electrical response [57]. The sonochemistry sample showed a lower resistance value, which could be related to the difference in grain size in the samples leading to a different proportion of grain boundaries [58,59].

**Figure 15.**
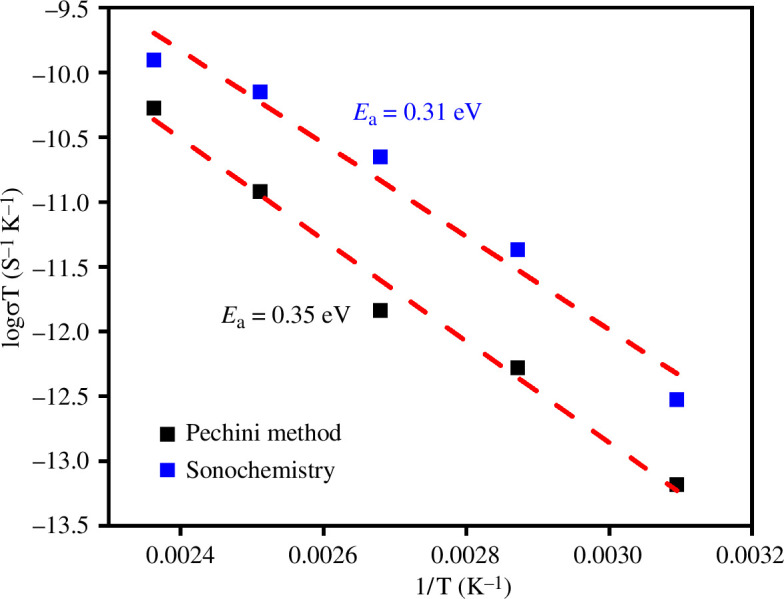
Activation energies (*E*_a_) of La_0.9_Sr_0.1_Fe_0.9_Co_0.1_O_3±*δ*_ obtained by the Pechini and sonochemistry methods.

From the impedance data, the conductivities of the single element observed within the frequency range were extracted (figure 15*a*,*c*). For both samples, from the magnitude of the capacitance, the electric response is attributed to bulk effects [60,61]. In particular, the LSFC system has been described by *R*–CPE elements, where *R* (the real part of the impedance) is a resistance and CPE is a constant phase element (see electronic supplementary material, file S1). From the impedance spectroscopy (IS) data, the *R* values were determined from the diameters of the semicircles, since the values correspond to the real part of the impedance [60]. The CPE element was used instead of a pure capacitor to suppress dispersion and non-linearities in the obtained values. Figure 14*b*,*d* shows the fitting data for the LSFC sample at 100°C in both samples: the calculated results for the equivalent circuits are presented in table 2. These RC components (resistor-capacitor) represent the macroscopic processes involved in charge transport due to lack of homogeneity in the microstructure. From the IS results it could be observed that when the radius of the semicircles decreased, the electric resistance decreased.

**Table 2.** Fitted data for the equivalent circuits of La_0.9_Sr_0.1_Fe_0.9_Co_0.1_O_3±_*_δ_*. for different elements: resistor (R), Constant Phase Elemen - quasicapacitance (CPE-T) and Constant Phase Element - arc slope (CPE-Q).

	Pechini method		Sonochemistry	
Element	value	% error	value	% error
*R* (Ω)	137 030	0.71	46 287	0.05
CPE-T (F)	9.174 E–12	1.28	9.414E–12	0.18
CPE-P (F)	1.003	0.08	0.999	0.01

The electrical conductivity (σ) for sintered materials follows the Arrhenius law, therefore the activation energies (*E*_a_) for both cases were calculated from the slope obtained from the Arrhenius equation (figure 15). For both synthesis methods, the activation energy was around 0.3 eV, which confirms the easy migration of charge carriers in LSFCs, which may be associated with high electron mobility. This confirms the UV–Vis results (figure 12), where the band of direct transitions describes small band gap values for both samples.

## 4. Conclusions

The use of the sonochemistry method allows La_0.9_Sr_0.1_Fe_0.9_Co_0.1_O_3±*δ*_ ceramic materials to be obtained with properties comparable to those prepared by the reported Pechini method. A cubic perovskite structure (*Pm*3̅*m* group (221)) and cell parameter *a* around 3.9 Å were determined for samples synthesized by both synthesis methods, Pechini and sonochemistry. These results were confirmed by XRD, Rietveld refinement and HRTEM. For the sample obtained by Pechini route, the crystal size and strain were 21 nm and 0.2%, respectively, while for the sample obtained by sonochemistry the crystal size was 10 nm with 1.4% strain, according to W–H graphs.

Furthermore, the optical analysis confirmed the semiconductor character of the samples, supported by the *E*_g_ values obtained; since the sonochemistry sample is the one with the smallest grain size, a greater energy was required for the activation of the sample. Considering the semiconductor character associated with the value of the band gap, porosity and grain size, the use of these materials in photocatalytic applications may be possible.

The obtained results are of great relevance in environmental applications since they determine the effective area of the material, either in fuel cells or as photodegradation catalysts, where it is important to reduce the size of the crystals to increase the active sites of the material. Furthermore, since these ceramic materials showed high symmetry, thermal stability, good porosity and low activation energy, they could also have potential use as a cathode in SOFC, opening the opportunity to further reduce the operating temperature of these devices.

## Data Availability

The datasets supporting this article have been uploaded as part of the online electronic supplementary material accompanying this article [62].
